# Piceatannol Protects Brain Endothelial Cell Line (bEnd.3) against Lipopolysaccharide-Induced Inflammation and Oxidative Stress

**DOI:** 10.3390/molecules27041206

**Published:** 2022-02-11

**Authors:** Yan Zhou, Haroon Khan, Maggie Pui Man Hoi, Wai San Cheang

**Affiliations:** 1 State Key Laboratory of Quality Research in Chinese Medicine, Institute of Chinese Medical Sciences, University of Macau, Macao SAR, China; yc07517@umac.mo (Y.Z.); maghoi@um.edu.mo (M.P.M.H.); 2 Department of Pharmacy, Abdul Wali Khan University, Mardan 23200, Pakistan; haroonkhan@awkum.edu.pk

**Keywords:** piceatannol, inflammation, oxidative stress, brain endothelial cells, lipopolysaccharide

## Abstract

Dysfunction of the blood–brain barrier (BBB) is involved in the pathogenesis of many cerebral diseases. Oxidative stress and inflammation are contributing factors for BBB injury. Piceatannol, a natural ingredient found in various plants, such as grapes, white tea, and passion fruit, plays an important role in antioxidant and anti-inflammatory responses. In this study, we examined the protective effects of piceatannol on lipopolysaccharide (LPS) insult in mouse brain endothelial cell line (bEnd.3) cells and the underlying mechanisms. The results showed that piceatannol mitigated the upregulated expression of adhesion molecules (ICAM-1 and VCAM-1) and iNOS in LPS-treated bEnd.3 cells. Moreover, piceatannol prevented the generation of reactive oxygen species in bEnd.3 cells stimulated with LPS. Mechanism investigations suggested that piceatannol inhibited NF-κB and MAPK activation. Taken together, these observations suggest that piceatannol reduces inflammation and oxidative stress through inactivating the NF-κB and MAPK signaling pathways on cerebral endothelial cells in vitro.

## 1. Introduction

Endothelial cells constitute the blood–brain barrier (BBB), which is the interface between the vasculature and the brain. The BBB maintains the homeostasis of the brain environment and the normal physiological functions of the central nervous system (CNS) by regulating the transfer of ions, molecules, and cells between the brain parenchyma and blood [1]. Its unique property is the prevention of unwanted substances from entering the brain via the blood, but damage to it will cause many pathological reactions [2]. Dysfunction of the BBB will lead to the entry of foreign harmful substances into the brain through the barrier and cause a variety of neurological diseases, such as Alzheimer’s disease, ischemic stroke, multiple sclerosis, and traumatic brain injury [3,4]. Cerebral microvascular endothelial cells (CMECs) are the main lumen components of the BBB [5]. Different from endothelial cells in non-neural tissues, CMECs are characterized by junction complexes, including tight junctions, adherens junctions, and gap junctions, strictly regulating the channel between the brain and blood [6].

Inflammation and oxidative stress result in BBB dysfunction. CMECs induce the recruitment and migration of leukocytes by upregulating cellular adhesion molecules, including intercellular adhesion molecule-1 (ICAM-1) and vascular cell adhesion molecule-1 (VCAM-1), causing local inflammatory responses and the secretion of pro-inflammatory regulators such as inducible nitric oxide synthase (iNOS) in the brain [7]. Oxidative stress causing BBB injury is closely related to the etiology and pathogenesis of brain diseases. Oxidative stress is caused by the imbalance between oxidants and antioxidants [8]. In the process of BBB injury, the production of enhanced reactive oxygen species (ROS) due to the activation of NADPH oxidase can destroy the normal functions of nerves and cells, leading to cell death [9]. Previous studies have supported the notion of crosstalk between inflammation and oxidative stress by enhancing the signal transduction of transcription factors such as nuclear factor-κB (NF-κB) and the expression of c-Jun N terminal kinase (JNK) and p38 mitogen-activated protein kinase (MAPK), contributing to the destruction of brain tissues [10]. Many stimuli, such as lipopolysaccharide (LPS), disrupt brain homeostasis and BBB function, resulting in a loss of tight junction proteins in CMECs, the secretion of pro-inflammatory cytokines, and the production of ROS [11]. LPS, as an endotoxin, causes organ damage, such as damage to brain tissue by inducing immune cells to produce many pro-inflammatory genes [12]. Peripheral LPS can activate the NF-κB and MAPK signaling pathways and cause oxidative stress and inflammatory response in CMECs in vitro [13]. Therefore, inhibiting LPS-induced inflammation and oxidative stress in CMECs is a potential treatment method for BBB dysfunction.

**Figure 1:**
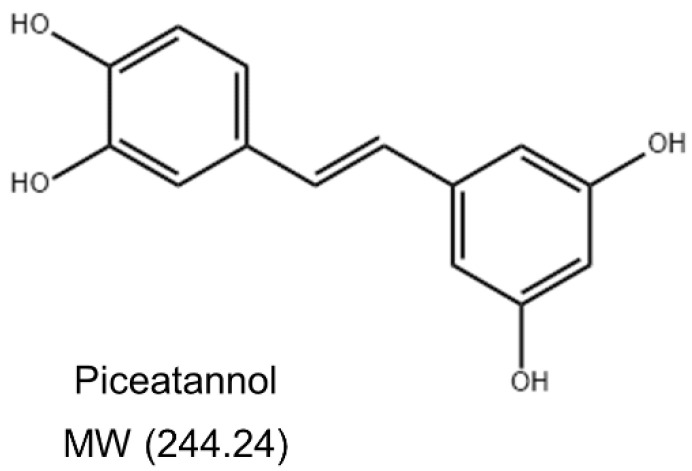
Structure of piceatannol.

Piceatannol (3,3′,4,5′-trans-trihydroxystilbene) (Figure 1) is a naturally occurring polyphenolic stilbene commonly found in various food such as grapes, white tea, and passion fruit [14]. Piceatannol has a wide range of biological activities, such as anti-cancer, antioxidant, anti-inflammatory, and immune regulatory activities [15]. Piceatannol was found to inhibit the activation of NF-κB induced by various inflammatory factors such as LPS in human bone marrow cells [16]. Piceatannol can effectively inhibit H_2_O_2_-induced DNA damage, indicating that it has a significant antioxidant effect on leukemia L1210, HL-60, and K562 cells [17]. In addition, piceatannol can suppress the allergic inflammatory response of mast cells induced by stimulants by regulating the secretion of pro-inflammatory cytokines and the expression of MAPK phosphorylation, indicating its potential therapeutic significance for allergic diseases [18]. The protective effect of piceatannol on endothelial cells has been extensively studied. Piceatannol enhances the NO bioavailability by increasing endothelial nitric oxide synthase (eNOS) expression and phosphorylation in endothelial cells [19]. Piceatannol can also act as a heme oxygenase-1 (HO-1) inducer by enhancing the HO-1 expression of in endothelial cells [20]. Piceatannol can ameliorate endothelial dysfunction by enhancing eNOS activity and the inhibition of oxidative stress [21]. Piceatannol possesses significant therapeutic potential against cardiovascular diseases [22]. However, whether piceatannol can prevent BBB damage and, in turn, neurological diseases remains to be explored, along with its molecular mechanism. This study investigated the protective effects of piceatannol against LPS-induced inflammation and oxidative stress in mouse brain microvascular endothelial cells (bEnd.3).

## 2. Results

### 2.1. Piceatannol Ameliorated LPS-Induced Adhesion Molecules (ICAM-1/VCAM-1) Protein Expression in bEnd.3 Cells without Affecting Cell Viability

**Figure 2:**
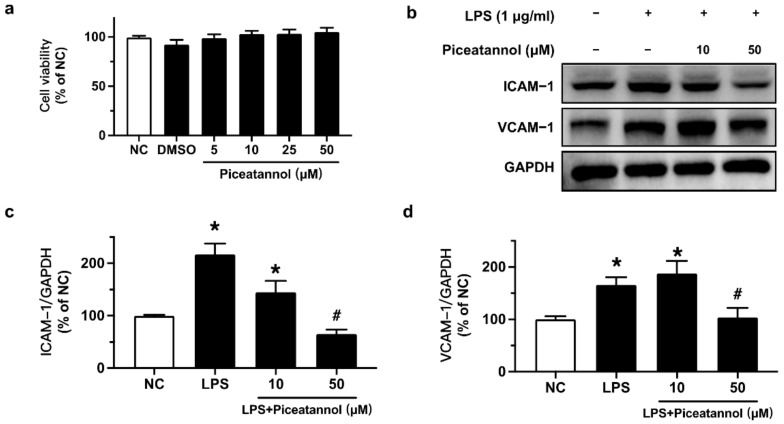
Effects of piceatannol on cell viability and LPS-induced adhesion molecules expression in bEnd.3 cells. (**a**) The effect of piceatannol on cell viability in bEnd.3 cells determined by MTT assay. (**b**) Representative blots and (**c**,**d**) summarized graph for ICAM-1 and VCAM-1 expression in cells pretreated with piceatannol (10, 50 µM) for 4 h and co-stimulated with LPS (1 μg/mL) for 24 h. Results are mean ± SEM, *n* = 3–4 experiments. * *p* < 0.05 vs. NC; # *p* < 0.05 vs. LPS.

Piceatannol’s effect on cell viability was measured by an MTT assay. bEnd.3 cells were exposed to LPS (10 μg/mL) for 24 h with or without piceatannol (10 and 50 μM). The results showed that the solvent, DMSO, and piceatannol (5, 10, 25, and 50 μM) had no significant effect on the cell viability on bEnd.3 cells (Figure 2a). Therefore, the effects of piceatannol at the safe concentrations of 10 and 50 µM were assessed on bEnd.3 cells stimulated with LPS (1 or 10 μg/mL).

To verify the inhibitory effects of piceatannol on LPS-induced adhesion molecules protein expression, we performed Western blotting. The protein expression of ICAM-1 and VCAM-1 was upregulated by LPS (1 μg/mL) exposure for 24 h, whereas such upregulation was reversed by pretreatment with 50 μM piceatannol for 4 h in bEnd.3 cells (Figure 2b–d).

### 2.2. Piceatannol Inhibited iNOS Expression and Proteins Relevant to MAPK Signaling Pathways in LPS-Treated bEnd.3 Cells

**Figure 3:**
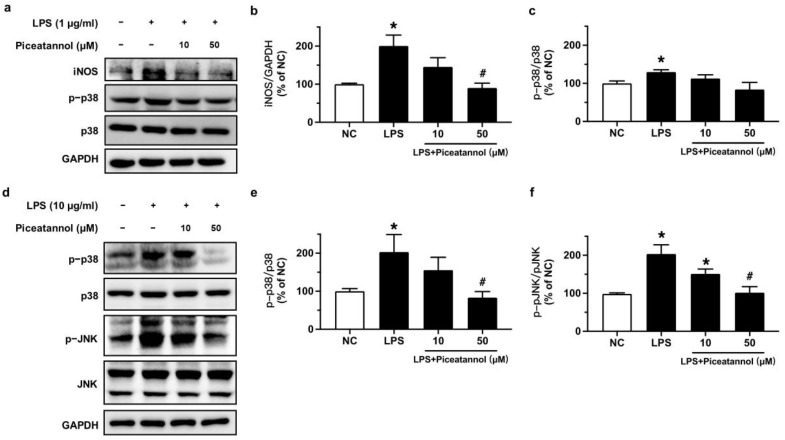
The effects of piceatannol on expression level of iNOS and MAPK signaling in bEnd.3 cells stimulated with LPS. (**a**) Representative blots and (**b**,**c**) summarized data for iNOS expression and the phosphorylation of p38 at Thr180/Tyr182 in cells pretreated with piceatannol (10 and 50 μM) for 4 h and co-incubation of LPS (1 μg/mL) for 24 h. (**d**) Representative blots and (**e**,**f**) summarized data for the phosphorylation of p38 (Thr180/Tyr182) and JNK (Thr183/Tyr185) in cells incubated with or without piceatannol (10 and 50 μM) for 30 min and then co-stimulated with LPS (10 μg/mL) for 1 h. Results are mean ± SEM, *n* = 3–4 experiments. * *p* < 0.05 vs. NC; # *p* < 0.05 vs. LPS.

To confirm the role of iNOS and MAPK signaling pathways in the protective effect of piceatannol in bEnd.3 cells stimulated with LPS, we performed Western blotting. Exposure to LPS (1 μg/mL) for 24 h increased iNOS expression while the pre-incubation of piceatannol for 4 h at 50 μM effectively inhibited the iNOS expression when normalized to GAPDH (Figure 3a,b). However, the phosphorylation of p38 at Thr180/Tyr182 was not altered in bEnd.3 cells with the same culture conditions (Figure 3a,c). The treatment of bEnd.3 cells with 10 μg/mL LPS for 1 h significantly increased the phosphorylation levels of p38 (Thr180/Tyr182) and JNK (Thr183/Tyr185) proteins. These changes were decreased by pretreatment with piceatannol for 30 min at 50 μM in comparison to the respective total protein (Figure 3d–f).

### 2.3. Piceatannol Suppressed NF-κB Inflammatory Signaling in bEnd.3 Cells upon LPS Stimulation

**Figure 4:**
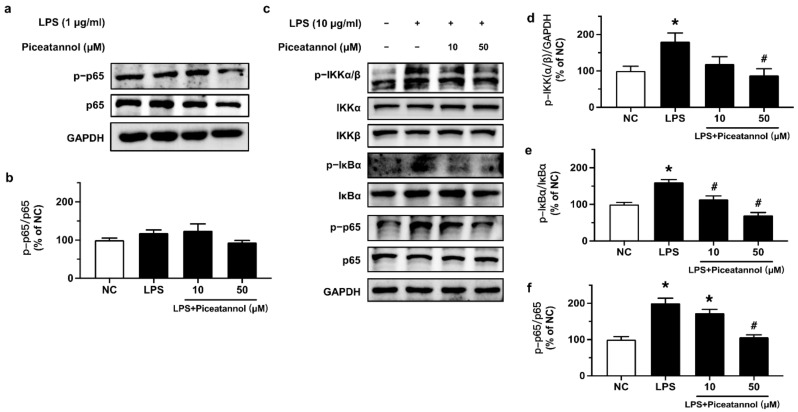
The effects of piceatannol on NF-κB inflammatory signaling in bEnd.3 cells upon LPS stimulation. (**a**,**b**) Phosphorylation level of p65 at Ser536 in bEnd.3 cells treated with piceatannol (10 and 50 μM, 4 h) followed by co-stimulation with 1 μg/mL LPS for 24 h. (**c**) Representative blots and (**d**–**f**) summarized data showing the effect of piceatannol on the phosphorylation of IKKα/β, IκBα and p65 in cells treated with or without piceatannol (10 and 50 μM) for 30 min and then co-stimulated with LPS (10 μg/mL) for 1 h. Results are mean ± SEM, *n* = 3–4 experiments. * *p* < 0.05 vs. NC; # *p* < 0.05 vs. LPS.

The effect of piceatannol on NF-κB signaling in bEnd.3 cells upon LPS stimulation was investigated by Western blotting. Cells were treated with 10 and 50 μM piceatannol for 4 h and then co-stimulated with 1 μg/mL of LPS for 24 h. Western blotting results showed a very minor and non-significant effect on the phosphorylation (Ser536) and expression of p65 (Figure 4a,b). Therefore, cells were stimulated with a higher concentration of LPS at 10 μg/mL for 1 h, which activated the NF-κB inflammatory signaling pathway through enhancing the phosphorylation of IKKα/β (Ser176/180), IκBα (Ser32), and p65 (Ser536). Pretreatment with piceatannol (10 and 50 μM, 30 min) significantly ameliorated p-IKKα/β, p-IκBα, and p-p65 in a concentration-dependent manner without any effect on the corresponding total protein expression (Figure 4c–f).

**Figure 5:**
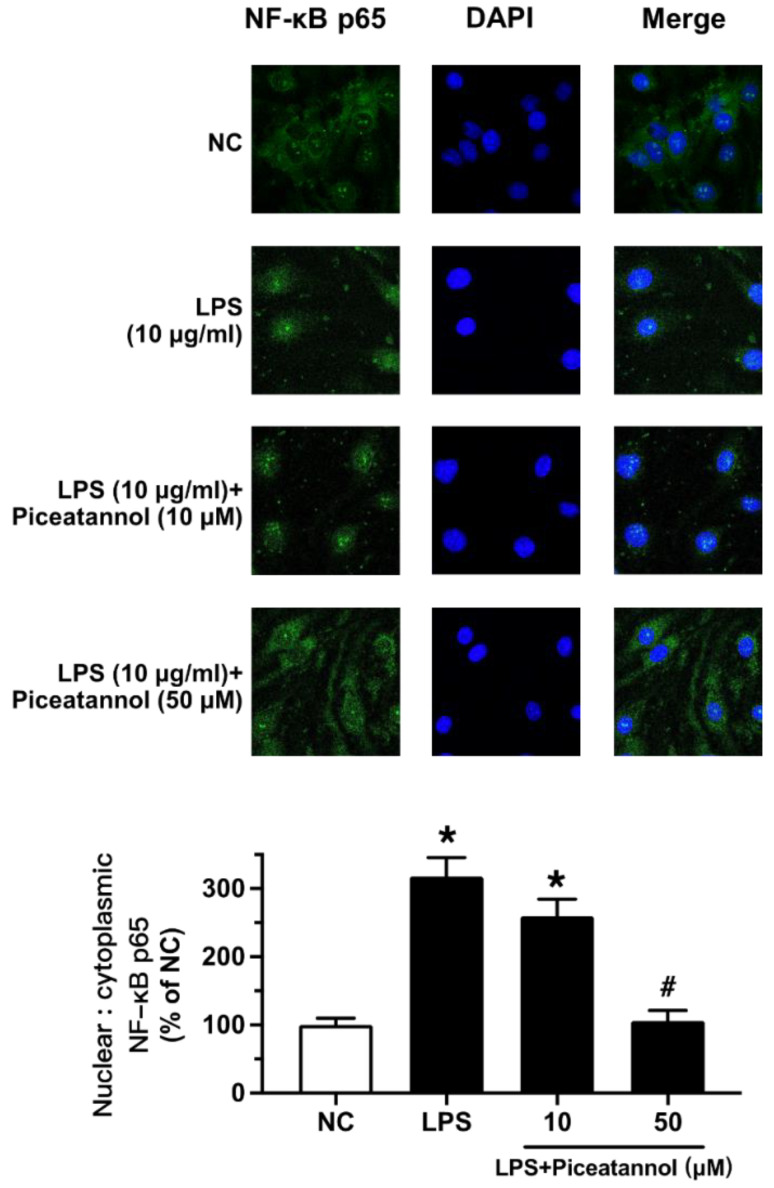
The effect of piceatannol on the LPS-induced nuclear translocation of NF-κB p65 in bEnd.3 cells. Representative images for the cells pretreated with different concentrations of piceatannol (10, 50 µM) for 30 min and then co-treated with LPS (10 μg/mL) for 1 h. NF-κB p65 (green) was represented by immunofluorescence staining and the nuclei were indicated by DAPI staining (blue). Results are mean ± SEM, *n* = 3 experiments. * *p* < 0.05 vs. NC; # *p* < 0.05 vs. LPS.

The effect of piceatannol on NF-κB signaling was further confirmed by fluorescence imaging. LPS exposure (10 μg/mL, 1 h) triggered NF-κB translocation from the cytosol to the nucleus of bEnd.3 cells. Such translocation was prevented by piceatannol pretreatment at 50 µM for 30 min (Figure 5).

### 2.4. Piceatannol Diminished LPS-Induced Oxidative Stress in bEnd.3 Cells

**Figure 6:**
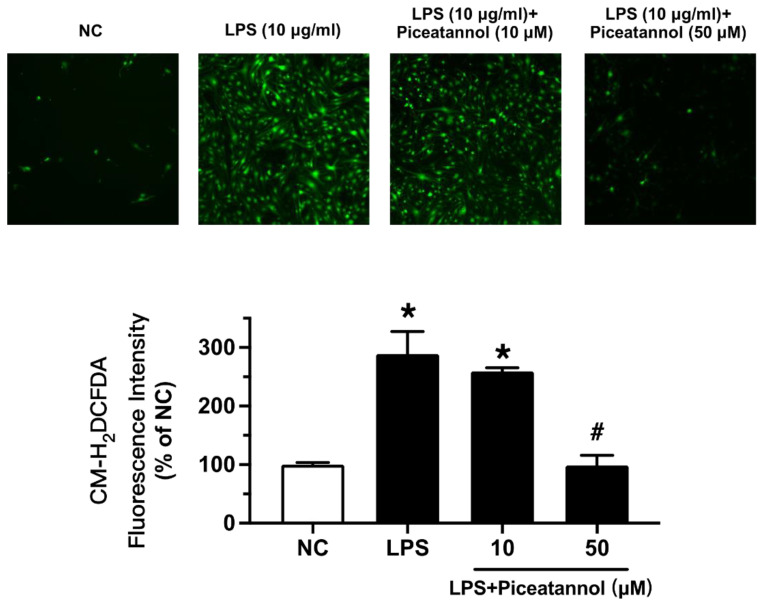
The effect of piceatannol on LPS-induced oxidative stress in bEnd.3 cells. After piceatannol (10, 50 µM) pretreatment for 30 min and then co-treatment with LPS (10 μg/mL) for 1 h, the ROS level in the bEnd.3 cells was visualized with CM-H_2_DCFDA incubation by fluorescence imaging. Results are mean ± SEM, *n* = 3 experiments. * *p* < 0.05 vs. NC; # *p* < 0.05 vs. LPS.

We examined whether piceatannol diminished ROS production in bEnd.3 cells with LPS stimulation using CM-H_2_DCFDA fluorescence dye. The ROS levels were enhanced by LPS exposure at 10 µg/mL for 1 h, while piceatannol pretreatment for 30 min reduced the ROS products, as indicated by CM-H_2_DCFDA fluorescence (Figure 6).

## 3. Discussion

The present study showed that piceatannol ameliorated inflammatory response and oxidative stress in LPS-treated bEnd.3 cells by the following results: (1) piceatannol at high concentration (50 µM) markedly suppressed the expression of adhesion molecules ICAM-1 and VCAM-1; (2) piceatannol reduced iNOS expression as well as inactivating the NF-κB and MAPK signaling pathways; and (3) piceatannol diminished ROS production.

LPS is widely used as an inducer of inflammation in various cells, including macrophages [23]. As an endotoxin, LPS enhances the release of pro-inflammatory mediators, leading to the destruction of brain tissue and BBB dysfunction [24]. iNOS, the most familiar pro-inflammatory cytokine, induces an inflammatory response by inducing the excessive production of NO [25]. LPS has also been reported to cause oxidative stress by increasing ROS generation [24]. ROS are closely associated with neurological diseases and are a key mediator in promoting inflammatory response and BBB destruction [26]. A number of experimental studies have shown that an excessive increase in ROS reduces the expression of tight junction proteins in endothelial cells, which increases BBB permeability and causes nervous-system-related diseases [27,28]. Therefore, a reduction in ROS production or enhancement of antioxidant activity in the body is a common therapeutic strategy. In this study, we found that the antioxidant piceatannol can reduce inflammatory responses and ROS levels in brain endothelial cells by regulating intracellular mechanisms.

Cell adhesion molecules, including ICAM-1 and VCAM-1, play an important role in neurological diseases [29]. Both ICAM-1 and VCAM-1 exist in vascular endothelial cells, mediating the process of pathological conditions such as brain injury inflammation. When LPS triggers an inflammatory response, the expression of adhesion molecules increases, resulting in leukocytes adhering to vascular endothelial cells, which increases the permeability of the vascular wall and destroys BBB function [30]. Extensive evidence shows that LPS can activate the MAPK and NF-κB signaling pathways and damage the central nervous system [31,32]. The MAPK signaling cascade is a major regulator involved in oxidative stress and inflammatory response and is also related to the protein expression of iNOS [33]. MAPKs are serine/threonine kinases, mainly including the p38 and c-JNK families [34]. An increasing number of studies have demonstrated that MAPKs play an important role in the central nervous system [32]. Our study found that piceatannol can effectively inhibit the LPS-induced activation of MAPK pathways in bEnd.3 cells. Moreover, NF-κB is a common transcription factor and a major mechanism of inflammation [35]. Studies have shown that the NF-κB pathway up-regulates the expression of adhesion molecules in bEnd.3 cells and enhances oxidative stress [36]. NF-κB mainly involves the IKKα/β, IκBα and p65 signaling pathways [37], which were determined in the present study to be a potential mechanism modulating the effect of piceatannol.

Piceatannol, a 3,3′,4,5′-trans-trihydroxystilbene, is commonly found in grapes [38]. Previous studies have demonstrated that piceatannol presents anti-cancer, antioxidant, and anti-inflammatory activities, and can effectively ameliorate endothelial dysfunction [19,39]. Piceatannol can inhibit the NF-κB and MAPK signaling pathways with its antioxidant activity and therefore prevent oxidative damage and inflammatory response in osteoclasts [40]. Piceatannol is used to treat a variety of pathological conditions, such as cardiovascular disease, Alzheimer’s disease, and sickle cell disease [22,41,42]. In this study, piceatannol was selected to explore its protective role in brain microvascular endothelial cells against inflammation and oxidative stress and the underlying molecular mechanism. We found that piceatannol effectively reduced the expression of LPS-induced adhesion molecules. Subsequently, the treatment of piceatannol downregulated the expression of LPS-activated iNOS, which verified the inhibitory effect of piceatannol on pro-inflammatory cytokines in neurodegenerative diseases. The Western blot results showed that piceatannol inhibited the MAPK signaling pathways in bEnd.3 cells upon LPS stimulation. Incubating with low concentrations of LPS (1 μg/mL) for 24 h, piceatannol did not significantly improve the phosphorylation of p38. This is consistent with previous studies that indicated that LPS at low concentrations and long exposure times was not able to induce MAPK signaling pathways or that the effect was absent in brain endothelial cells [7,43]. Adjusting the treatment conditions to the pretreatment of piceatannol (10 and 50 μM, 30 min) followed by the co-stimulation of LPS (10 μg/mL, 1 h), piceatannol notably diminished the phosphorylation levels of p38 and JNK MAPKs. On the other hand, piceatannol acts on the NF-κB signaling cascade. Piceatannol at 50 μM strongly suppressed the phosphorylation of p65, IKKα/β, and IκBα in the NF-κB signaling pathway. Likewise, piceatannol blocks NF-κB p65 subunit translocation to the nucleus of bEnd.3 cells.

Of note, LPS-induced ROS production can activate various signaling pathways and is closely associated with a variety of diseases [44]. CM-H_2_DCFDA is oxidized by intracellular H_2_O_2_ into dichlorouorescein (DCF) and can be used as a fluorescent probe to measure the level of ROS production in LPS-stimulated bEnd.3 cells [45]. We found that LPS (10 μg/ mL, 1 h) increased ROS levels, which were suppressed by pretreatment with piceatannol at 50 μM for 30 min. These experimental results showed that piceatannol significantly reduced LPS-induced inflammation and oxidative stress in brain endothelial cells, indicating potential therapeutic significance against BBB dysfunction.

Our present findings are in line with previous reports showing that inflammatory and oxidative stress responses are involved in LPS-induced damage, causing the downregulation of tight junctions and adherens junctions, an increase in BBB permeability, and thus BBB disruption [24], as well as reports that anti-inflammatory and antioxidant drugs—for example, metformin [46] and platonin [47]—can upregulate tight junction proteins to protect BBB function. Herein, we suggest that piceatannol can help in preventing hyperpermeability in endothelial cells, possibly through the suppression of the MAPK and NF-κB signaling pathways and the inhibition of oxidative stress. However, other direct or indirect mechanisms such as mitochondrial function and expression of tight junctions and adherens junctions are to be examined in the future. The piceatannol analogs, pterostilbene and resveratrol, also exhibit anti-inflammatory and antioxidant activities, but are less active than piceatannol. In vascular endothelial cell models with elevated oxidative stress, piceatannol was found to have a stronger activity in restoring dimethylarginine dimethylaminohydrolase of endothelial cells than resveratrol [21]. Additionally, piceatannol showed a stronger anti-inflammatory effect than resveratrol in terms of inhibiting the release of NO and prostaglandin E2 [48]. More interestingly, piceatannol has a protective effect against DNA damage, while pterostilbene and resveratrol aggravated DNA damage [49].

Many studies on the bioactivity of piceatannol have reported its anti-inflammatory and/or antioxidative activities [50,51,52]. In line with our current findings, piceatannol exhibits potential for clinical development. Unfortunately, the beneficial effects of antioxidant treatment remain controversial, as antioxidants exhibit disappointing results in terms of disease progression and severity in a clinical setting. Piceatannol has been greatly limited in clinical applications due to its poor liberation characteristics. Therefore, scientific investigations are trying to overcome this obstacle through different approaches. For instance, nanotechnology is a possible approach to prepare drug vehicles for the controlled release of piceatannol [53]. Further investigations are essential to confirm the effectiveness of piceatannol in terms of clinical application and to find a suitable modality for optimizing its effect.

## 4. Materials and Methods

### 4.1. Chemicals and Reagents

Piceatannol was purchased from Tokyo Chemical Industry (Tokyo, Japan). Dulbecco’s modified Eagle’s medium (DMEM) was bought from GE Healthcare Life Sciences HyClone Laboratories (Logan, UT, USA), while fetal bovine serum (FBS) and penicillin plus streptomycin (PS) were obtained from Gibco (Carlsbad, CA, USA). 3-(4,5-dimethylthiazol-2-yl)-2,5 diphenyl tetrazolium bromide (MTT) and LPS were purchased from Sigma-Aldrich (St. Louis, MO, USA). The secondary antibodies, RIPA buffer, phenyl methane sulfonyl fluoride (PMSF), Protease Inhibitor Cocktail, BCA protein assay kit for Western blotting, 4% paraformaldehyde (PFA), Alexa Fluor 488-labeled secondary antibody, and 2-(4-amidinophenyl)-6-indolecarbamidine dihydrochloride (DAPI) were acquired from Beyotime biotechnology (Shanghai, China), while all the primary antibodies were obtained from Cell Signaling Technology (Danvers, MA, USA). PVDF membranes were acquired from Millipore (Billerica, MA, USA). 5-(and-6)-chloromethyl-2′,7′-dichlorodihydrofluorescein diacetate acetyl ester (CM-H_2_DCFDA) was purchased from Molecular Probes (Eugene, OR, USA).

### 4.2. Cell Culture

Cells from bEnd.3, a mouse brain endothelial cell line, were purchased from the American Type Culture Collection (ATCC) (Manassa, VA, USA). The bEnd.3 cells were cultured in DMEM containing 10% FBS plus 100 U/mL penicillin and 100 U/mL streptomycin at 37 °C in a humidified atmosphere with 5% CO_2_. Cells were subcultured every 2–3 days. All the experiments with bEnd.3 cells were performed between passages 5 to 25 for maintaining excellent BBB characteristics in vitro [54]. The bEnd.3 cell impaired model was induced by treating with LPS at two different concentrations and stimulation times (1 μg/mL for 24 h or 10 μg/mL for 1 h) to examine different target proteins modulating inflammatory and antioxidant responses in accordance with previous literature [24]. Piceatannol (10 or 50 μM) was added to the medium for 30 min and then LPS (10 μg/mL) was added in the existing medium containing piceatannol for 1 h. For another set of experiments, cells were pretreated with piceatannol for 4 h before LPS stimulation (1 μg/mL for 24 h) in the existing medium. Piceatannol was dissolved in 1% dimethyl sulfoxide (DMSO), and LPS was dissolved in phosphate-buffered saline (PBS).

### 4.3. Cell Viability Assay

The cell viability was evaluated by MTT assay. bEnd.3 cells (5 × 10^4^/well) were seeded in 96-well plates and incubated at 37 °C with 5% CO_2_ overnight and then treated with different concentrations of piceatannol for 24 h. After the incubation, the medium was refreshed with the one containing 10% MTT reagent for another 4 h. The MTT medium was replaced with 100 µL DMSO in each well to dissolve the formazan crystals. The absorbance at 570 nm was read using the SpectraMax M5 microplate reader (Molecular Devices, Silicon Valley, CA, USA).

### 4.4. Western Blotting Analysis

bEnd.3 cells (7.5 × 10^5^/well) were seeded in 6-well plates and incubated at 37 °C with 5% CO_2_ overnight. The western blotting analysis was divided into two groups: one group of bEnd.3 cells were incubated with 10 μM and 50 μM piceatannol for 4 h, followed by 24 h LPS (1 μg/mL) stimulation. Another group of cells were pretreated with different concentrations of piceatannol for 30 min before exposure to LPS (10 μg/mL) for 1 h. The cells were lysed with RIPA buffer supplemented with 1% PMSF and 1% Protease Inhibitor Cocktail on ice for 10 min. After centrifugation at 15,000 rpm for 15 min at 4 °C, the supernatants were collected and the protein concentration was determined by BCA protein assay kit. Equal amounts of protein sample (20 μg) were separated by 10% SDS/PAGE and transferred onto PVDF membranes. Subsequently, the membranes were blocked by 5% non-fat milk in 0.05% Tween-20 PBS and incubated with respective primary antibodies against ICAM-1 (#4915), VCAM-1 (#13662), iNOS (#13120), p-p38 (#4511), p38 (#8690), p-JNK (#9255), JNK (#9253), p-IKKα/β (#2697), IKKα (#2682), IKKβ (#2678), p-IκBα (#2859), IκBα (#4814), p-p65 (#3033) and p65 (#8242) (1:1000) (Cell Signaling Technology) overnight at 4 °C. GAPDH (#5174) (1:5000) was selected as housekeeping protein for checking equal loading of each sample. After washing with 0.05% Tween-20 PBS, the membranes were incubated with the corresponding secondary antibodies (1:1000) (anti-rabbit, A0208; anti-mouse, A02616, Beyotime) for 2 h at room temperature. The protein bands were visualized with an enhanced chemiluminescence (ECL) system (GE Healthcare, Pittsburgh, PA, USA) and scanned by ChemiDoc MP Imaging System (Bio-Rad Laboratories, Hercules, CA, USA).

### 4.5. Intracellular ROS Measurement

The intracellular ROS level in each group was detected by CM-H_2_DCFDA. bEnd.3 cells (2.5 × 10^5^/well) were seeded in 24-well plates and incubated at 37 °C with 5% CO_2_ overnight. After piceatannol pretreatment for 30 min and co-treatment with LPS (10 μg/mL) for 1 h, the bEnd.3 cells were washed with PBS and incubated with CM-H_2_DCFDA (5 μM)-containing normal physiological saline solution (NPSS) at 37 °C for 30 min in dark conditions. The NPSS contains (mM): 140 NaCl, 5 KCl, 1 CaCl_2_, 1 MgCl_2_, 10 glucose, and 5 HEPEC (PH 7.4). Then, the dye was removed and washed three times with PBS. The fluorescent signals of the cells were measured using the Leica-DMi8 Inverted fluorescent microscope at an excitation of 475 nm and an emission of 590 nm (Leica Microsystems, Wetzlar, Germany). Densitometry analysis was measured by the Image-Pro Plus 6.0 software.

### 4.6. Immunofluorescence Assay

bEnd.3 cells (5 × 10^5^/confocal dish) cultured in a laser confocal Petri dish were pretreated with piceatannol for 30 min, followed by the co-incubation of LPS (10 μg/mL) for 1 h. The bEnd.3 cells were then washed by PBS, fixed with 4% PFA for 20 min, permeabilized with 1% Triton X-100 for 10 min and blocked with 3% BSA for 1 h at room temperature. Subsequently, the cells were incubated with primary antibody against NF-κB p65 (1:500) (#8242, Cell Signaling Technology) overnight at 4 °C, followed by incubation with Alexa Fluor 488-labeled secondary antibody (1:100) (A0423, Beyotime) for 2 h at room temperature. Finally, the cells were washed with PBS and subjected to DAPI incubation. The fluorescence images were captured by a confocal laser scanning microscope (Leica Microsystems, TCS SP8, Mannheim, Germany). The quantification (measurement of intensity) was performed using the Image-Pro Plus 6.0 software.

### 4.7. Statistical Analysis

All data were expressed as mean ± standard error of mean (SEM) of n separate experiments. Numerical data were compared using Student’s *t*-test or one-way ANOVA followed by Bonferroni post hoc tests for more than two treatments. All data were analyzed using the GraphPad Prism software (GraphPad Software, San Diego, CA, USA). The value of *p* < 0.05 was considered statistically significant.

## 5. Conclusions

In conclusion, our results revealed that piceatannol inhibits LPS-induced oxidative stress and inflammatory response in bEnd.3 cells by suppressing the NF-κB and MAPK signaling pathways and downregulating the expression of adhesion molecules (ICAM-1 and VCAM-1), all accomplished with the diminishment of ROS generation. Considering the beneficial effects of piceatannol on brain endothelial cells, it is necessary to further study the effect of piceatannol in vivo to verify its potential therapeutic significance for neurological diseases.

## Data Availability

The data presented in this study are available on request from the corresponding author.
